# A novel, highly sensitive, one-tube nested quantitative real-time PCR for *Brucella* in human blood samples

**DOI:** 10.1128/spectrum.00582-23

**Published:** 2023-10-04

**Authors:** Li Liu, Mengyao Li, Gaowen Liu, Jian He, Yang Liu, Xuesong Chen, Yungui Tu, Jie Lin, Yue Feng, Xueshan Xia

**Affiliations:** 1 Faculty of Life Science and Technology, Kunming University of Science and Technology, Kunming, Yunnan, China; 2 Yunnan Kecan Biotechnology Co., Ltd, Kunming, China; 3 The Affiliated Anning First People’s Hospital, Kunming University of Science and Technology, Kunming, China; 4 Yunnan SCISPARK Genetic Testing Lab, Yunnan SCISPARK Biotechnology Co., Ltd, Kunming, China; Taichung Veterans General Hospital, Taichung, Taiwan

**Keywords:** brucellosis, *bcsp31 *gene, one-tube nested qPCR, conventional qPCR

## Abstract

Brucellosis is a zoonotic disease caused by *Brucella* spp. and is a major threat to human and livestock health. The accurate and rapid detection of *Brucella* DNA is essential for the diagnosis and treatment of this disease. However, traditional diagnostic methods, such as culture and serological tests, have limitations in terms of sensitivity, specificity, and time consumption. To address these challenges, we developed a highly sensitive one-tube nested quantitative real-time PCR (qPCR) method to detect *Brucella* DNA using a closed-tube approach with two primers and two probes that sequentially react with encoding a *Brucella* outer membrane protein. The analytical sensitivity of this one-tube nested qPCR approach was 100 fg/μL, which is 100-fold higher than that of a conventional qPCR. Intra-batch and inter-batch replicates showed low coefficients of variation, both less than 5%. The study included 250 clinical samples, showing that this one-tube nested qPCR method has a specificity of 100% and a sensitivity of 98.6%, exceeding the sensitivity of conventional qPCR (84.1%) at a cycle threshold (CT) cutoff of 38. More importantly, the one-tube nested qPCR reduced the CT values by an average of 6.4 compared to conventional qPCR, significantly improving the detection rate of low-load samples (CT > 35). In conclusion, the *bcsp31* one-tube nested qPCR method is a promising tool for the detection of brucellosis, owing to its high sensitivity and operational simplicity. This study highlights the potential of qPCR-based methods to improve the accuracy and speed of brucellosis diagnoses.

**IMPORTANCE:**

This study developed a highly sensitive and efficient method for the detection of brucellosis by introducing a one-tube nested quantitative real-time PCR (qPCR) approach, representing a remarkable advance in the field. The method demonstrated an impressive analytical sensitivity of 100 fg/μL, surpassing conventional qPCR and enabling the detection of even low levels of *Brucella* DNA. In addition, the study’s comprehensive evaluation of 250 clinical samples revealed a specificity of 100% and a sensitivity of 98.6%, underscoring its reliability and accuracy. Most importantly, the new method significantly improved the detection rate of low-burden samples, reducing cycle threshold values by an average of 6.4. These results underscore the immense potential of this approach to facilitate rapid and accurate brucellosis diagnosis, which is critical for effective disease management and control.

## INTRODUCTION

Brucellosis is a severe infectious disease caused by the *Brucella* species that can lead to symptoms such as fever, headache, and joint pain (1, 2) and is endemic in more than 170 countries and regions (3). According to incomplete statistics, over a 1,000 new cases of human brucellosis are reported each year and the cumulative number of cases worldwide continues to rise (4, 5). China is also seriously threatened by brucellosis, with 69,767 brucellosis cases nationwide by 2021, according to the National Health Commission of the People’s Republic of China. Areas reporting cases are mainly concentrated in the north-east and north-west, but in recent years, these have tended to spread southwards, including the Yunnan Province, which had double the number of patients in 2021 (699) compared to that in 2020 (381), and the number of patients is increasing every year. Human brucellosis not only causes tremendous suffering to patients but also causes huge economic losses to people in affected areas, owing to expensive health care costs and prolonged inability to work.

*Brucella* spp., the causative agents of brucellosis, are small coccobacillary (0.5–1.5 μm), intracellular-extracellular pathogens, Gram-negative bacteria, and their infections cause clinical symptoms such as malaise, prolonged recurrent fever, arthralgia, excessive sweating, and enlargement of the liver, spleen, and lymph nodes (6, 7). According to the classification established by the Food and Agriculture Organization and World Health Organization expert committee on brucellosis in 1970, *Brucella* is divided into six classic species based on host preference, and 19 subtypes based on biological characteristics. These species include *B. melitensis* (subtypes 1, 2, and 3), *B. abortus* (subtypes 1, 2, 3, 4, 5, 6, 7, and 9), *B. suis* (subtypes 1, 2, 3, 4, and 5), *B. canis*, *B. neotomae*, and *B. ovis*. In addition, six new species have been recently discovered, namely, *B. microti*, *B. pinnipedialis*, *B. papionis*, *B. ceti*, *B. vulpis*, and *B. inopinata* (8, 9).

*Brucella* infections cause clinical symptoms that are heterogeneous, non-specific, and often associated with poor prognosis (10). If misdiagnosed and not treated promptly, the incidence of complications can increase, causing irreparable damage or even death. Complications such as spondylitis, peripheral arthritis, epididymitis, meningitis, bronchitis, and interstitial pneumonia can occur. Therefore, early diagnosis and treatment are essential to slow disease progression. *Brucella* is diagnosed through culture-based isolation from blood, bone marrow, or other tissue samples; however, this method has serious drawbacks, with a low detection sensitivity of approximately 50% in the acute phase of infection (11, 12). In addition, culture takes 4 days, and the harsh culture conditions are not conducive to a rapid diagnosis (12, 13). Serological diagnosis is not only ineffective in differentiating current form recurrent infections, but also is associated with antibody cross-reactivity with *Stenotrophomonas maltophilia*, *Escherichia coli O157:H7*, *Yersinia enterocolitica*, *Francisella tularensis* and *Bartonella*, which limits its clinical diagnostic value (11, 14). Polymerase chain reaction (PCR) is an important tool for the rapid diagnosis of *Brucella* infection (3, 15, 16). Quantitative real-time PCR (qPCR) has become an essential technology in clinical diagnostics because of its increased specificity and sensitivity compared to those of culture and serological tests, as well as its simplicity and time-saving operations (17). The two-step nested RT-PCR assay is sufficiently sensitive but time-consuming and susceptible to cross-contamination during the experiment. Owing to the shortcomings of available diagnostic methods, a rapid, sensitive, and specific molecular diagnostic method is of crucial importance.

The screening of *Brucella*-specific genes determines the sensitivity of detection methods based on qPCR technology with respect to the detection of *Brucella*. To date, specific primers and probes have been designed for the detection of *Brucella*, with most studies focusing on *bcsp31*, *16S rRNA*, *IS711*, *BMEI1162*, *BMEII0466*, *alkB*, *eryC*, and *per* (18 – 23). As the *bcsp31* is a single-copy gene of the *Brucella* genome, it can more accurately reflect the DNA load of this genus in the patient’s blood (24) and has been shown in several studies to confer higher assay sensitivity than other genes, such as *IS711* (25).

Therefore, in this study, we established a rapid and highly sensitive one-tube nested qPCR assay targeting the sequence encoding the conserved immunogenic membrane protein 31 kDa (BCSP31) of *Brucella*. The assay was based on conventional qPCR using a designed external primer. In an evaluation of clinical samples, the one-tube nested qPCR could decrease the cycle threshold (CT) value by an average of 6.4 and had a better detection rate for those samples with low *Brucella* DNA concentration (CT > 35). This is a novel and effective method for *Brucella* detection and has important potential for application.

## MATERIALS AND METHODS

### Primer and probe design

Primer probes were selected with specificity for the *bcsp31* sequence in the conserved region of *Brucella* spp. using the online PrimerQuest tool at https://sg.idtdna.com/PrimerQuest/Home/Index. Subsequently, sequence alignment analysis was performed using BLAST (http://www.ncbi.nlm.nih.gov/BLAST/) to ensure primer and probe specificity. All primers and probes were tested for dimer and hairpin formation using Oligo7. The fluorescent probes bcsp31-P and GAPDH-P are labeled with unique fluorescent reporter dyes (6-carboxyfluorescein [FAM]) and (indocarbocyanine [Cy5]) at the 5′ end and with Black Fluorescent Quencher (BHQ) at the 3′ end, respectively. The primers were synthesized by Tsingke Biotechnology Co., Ltd (Beijing, China), the probes were synthesized by Taihe Biotechnology Co., Ltd (Beijing, China), and the primer sequence information is shown in Table 1.

**TABLE 1:** Primer and probe used in one-tube nested qPCR and conventional qPCR assay

Primer name	Sequence 5′–3′	Product size (bp)	Reference
Bru-bcsp31-WF	CGCGTGCTTCAGGTGCG	260	This study
Bru-bcsp31-WR	CGTGCCGGGTCGTCGCGAC		
Bru-bcsp31-NF	GGGTAAAGCGTCGCCAGAAG	150	(26)
Bru-bcsp31-NR	GCGGTTGCCAATATCAATGC		
Bru-bcsp31-P	FAM-AAATTCCACTGCCTGCCATCA-BHQ1		
GAPDH-F	GAAGGTGAAGGTCGGAGTC	223	This study
GAPDH-R	GAAGATGGTGATGGGATTTC		
GAPDH-P	Cy5-ACGGATTTGGTCGTATTGGGC-BHQ1		

### *Brucella* blood culture and RBPT assay

A 10 mL blood specimen was collected from a patient with *Brucella* and incubated using traditional blood culture methods (27). Briefly, the blood samples were centrifuged at 2,000 rpm for 15 min to remove the serum. The sediment was then collected into a sterile screw-capped plastic tube containing glass beads and shaken on a shaker for 15 min to rupture the clot. The ruptured clots were then inoculated into Castaneda’s medium and incubated at 37°C with 10% CO_2_ for 28 days. The isolated bacteria were identified using microbiological methods, including Gram staining and biochemical tests such as urease, oxidase, and catalase. The *Brucella* rose Bengal plate (RBPT) was purchased from the China Veterinary Drug Administration, and experiments were conducted in a biosafety level 2 laboratory at the affiliated Anning First People’s Hospital, Kunming University of Science and Technology, in compliance with biosafety regulations.

### Preparation of *Brucella* positive standards

*Brucella* genomic DNA was used as a positive standard in the study. Briefly, the AxiPrep Blood Genomic DNA Medium Volume Kit was used to extract *Brucella* isolates. Clinical isolates were provided by the affiliated Anning First People’s Hospital, Kunming University of Science and Technology. PCR amplification of 16S rRNA target genes was performed using specific *16S rRNA* primers (27F/1492R). PCR amplification products were analyzed by 1% agarose gel electrophoresis for the identification of clinical isolates. The extracted *Brucella* genomic DNA was measured for concentration using Qubit 2.0 (ThermoFisher Scientific, China) and then the copy number corresponding to the *Brucella* positive standards was calculated according to the following formula:


$$Copy\;number=\frac{Concentration\left(\frac{ng}{\mu L}\right)\times Avogadro\;constant\left(NA\right)\times 10^{\text{-}9}}{660\times base\;number\;of\;Brucella\;genomic}$$

### One-tube nested qPCR assay and optimization of cycling conditions

In this study, based on previous studies, we designed external primers for *Brucella* 31 kDa outer membrane proteins and performed multiple rounds of one-tube nested qPCR assays to optimize their optimal primer working concentration, annealing temperature, and cycling conditions. Each one-tube nested qPCR assay was performed in a 25-µL system containing 12.5 µL of 2× Pro Taq HS Probe Premix, 0. 8 µL Bru-bcsp31-WF/WR (10 µM), 1 µL Bru-bcsp31-NF/NR (10 µM), 1 µL GAPDH-F/R (10 µM), 1 µL Bru-bcsp31-P and GAPDH-P (5 µM), 1.9 µL RNase-free water, and 3 µL DNA template. The one-tube nested qPCR assay was divided into two amplification steps performed under the following conditions: (i) the first amplification step included an initial denaturation at 95°C for 30 s, followed by 10 cycles of 95°C for 5 s and 68°C for 30 s and (ii) the second amplification step included 45 cycles of denaturation at 95°C for 5 s and annealing/extension at 58°C for 40 s. During the second amplification step, the fluorescence emission signal was detected at the end of each run. The single-tube nested qPCR assays were performed using the TaqMan system (CFX Connect Real-Time System, Bio-Rad, USA) and CT values were automatically analyzed in the CFX Connect Real-Time System.

### Analytical specificity, sensitivity, and reproducibility of one-tube nested qPCR assays

The optimal reaction conditions for one-tube nested qPCR experiments have been optimized. To better evaluate the method, we performed an assessment of analytical specificity and sensitivity. We evaluated the specificity of one-tube nested qPCR using six common pathogens with similar clinical signs to *Brucella* infection, including *Escherichia coli*, *Bacillus subtilis*, *Pseudomonas aeruginosa*, *Staphylococcus aureus*, *Streptococcus pneumoniae*, and *Haemophilus influenzae*. The *Brucella* genomic DNA was diluted in a gradient of 1 × 10^3^ ng/µL to 1 × 10^−4^ ng/µL and subjected to single-tube nested qPCR analysis for sensitivity testing. A standard curve was plotted based on CT and DNA concentration. The reproducibility of one-tube nested qPCR was evaluated using three concentrations of *Brucella* genomic DNA (10^3^ ng/µL, 10^2^ ng/µL, and 10^1^ ng/µL). Intra-assay and inter-assay reproducibilities of one-tube nested qPCR were tested using three concentrations of *Brucella* genomic DNA in three replicates and on three different days within 1 week. The formula for calculating the coefficient of variation (CV) is as follows:


$$CV\left(\%\right)=\frac{\;Standard\;deviation\;\left(SD\right)}{mean}\times 100\%$$

### Clinical samples

The clinical diagnostic criteria for this study comprised two parts: (i) epidemiological history, which included a history of close contact with animals bearing *Brucella* or their byproducts, as well as contact with people residing in areas where brucellosis is prevalent, and (ii) clinical manifestations, which included symptoms such as fever, malaise, excessive sweating, muscle and joint pain, and physical manifestations like an enlarged liver, spleen, lymph nodes, and testes. In addition, a positive primary immunological screening test was also considered. All 145 specimens were collected from patients who had been diagnosed with brucellosis and met both criteria i and ii. These whole blood samples were stored at approximately 4°C during transport and −80°C prior to analysis. DNA was extracted from *Brucella* nucleic acid using the AxiPrep Blood Genomic DNA Medium Volume Kit and tested using the one-tube nested qPCR assay; concurrent with the conventional qPCR assay.

## RESULTS

### Determination of positive standards

Target gene and 900 bp sequence information were successfully obtained from the *Brucella* isolates via 16S rRNA amplification and sequencing (Fig. S1A and B). The sequences were analyzed using NCBI BLAST and showed 100% similarity to that of the *Brucella melitensis* strain, sequence ID MT611105.1, which was finally identified as *Brucella melitensis* isolate (Fig. S1C).

### Primers and probes designed for one-tube real-time qPCR

**Fig 1:**
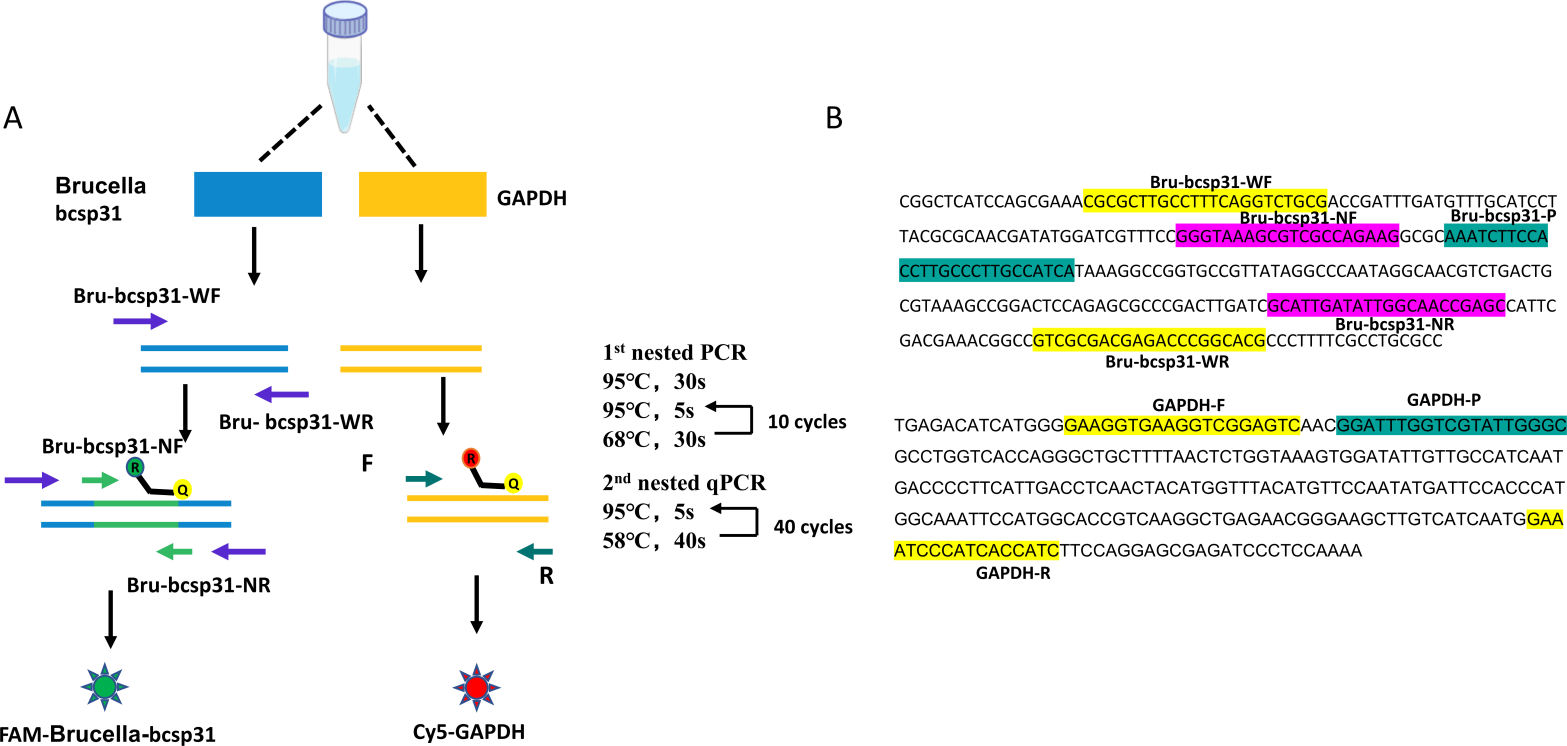
Schematic diagram of how one-tube nested qPCR works and primer/probe sequences. (A) Schematic diagram of how single-tube nested qPCR works. In the first round of PCR, a 260-bp fragment was amplified using external primers (Bru-bcsp31-WF and Bru-bcsp31-WR). The first round of PCR amplified for 10 cycles, generating a larger number of amplicons which were then used as a template for the second round of qPCR. A second pair of primers and probes (Bru-bcsp31-NF, Bru-bcsp31-NR, and Bru-bcsp31-P) was combined within the first amplicon to produce a shorter fragment (150 bp). Second round amplicons were detected with an FAM-labeled probe (495–520 nm). The internal control is only co-amplified with target *GAPDH* with GAPDH-F, GAPDH-R, and GAPDH-P at the second round of PCR, and the products were detected with a Cy5-labeled probe (646–662 nm). (B) The one-tube nested qPCR amplicon of *bcsp31* from *Brucella* and GAPDH nucleotide sequences are shown.

Previously, we designed primer probes for the detection of *Brucella bcsp31*. Here, we designed an outer primer pair, Bru-bcsp31 WF/Bru-bcsp31 WR, which covers the *bcsp31* amplicon and amplifies a larger fragment in the first round of amplification reactions. The amplicons generated using these primers in the first round of PCR were used as DNA templates for the second round of qPCR reactions. Additionally, we designed primer/probe sets targeting *GAPDH* as an internal quality control. For use in one-tube nested qPCR, the Bru-bcsp31 WF/WR primer pair was designed to have a higher melting temperature (Tm = 68°C) than that of the Bru-bcsp31 NF/NR primer pair (Tm = 58°C). Fluorescent probes with different fluorescence and burst motifs were designed based on the *bcsp31* and *GAPDH* genes. FAM-labeled *bcsp31* probes emitted green fluorescence and cy5-labeled *GAPDH* probes emitted red fluorescence(Table 1; Fig. 1B).

In a preliminary analysis, we optimized the melting temperature (Tm) and working concentration of the primer pairs to ensure the most efficient reaction conditions for amplification (Fig. S2). Ultimately, we determined the optimal reaction conditions as follows: the first amplification step included an initial denaturation at 95°C for 30 s, followed by 10 cycles of denaturation at 95°C for 5 s and annealing/extension at 68°C for 10 s. The second amplification step included 40 cycles of denaturation at 95°C for 5 s, after which the temperature was lowered to 58°C for 40 s for annealing/extension to allow for Bru-bcsp31-NF/NR and GAPDH-F/R binding and amplification (Fig. 1A). In summary, we have successfully established primer/probe pairs and amplification reaction procedures to detect the *Brucella bcsp31*.

### Validation of specificity and sensitivity of one-tube nested qPCR assay showed higher sensitivity compared to conventional qPCR

**Fig 2:**
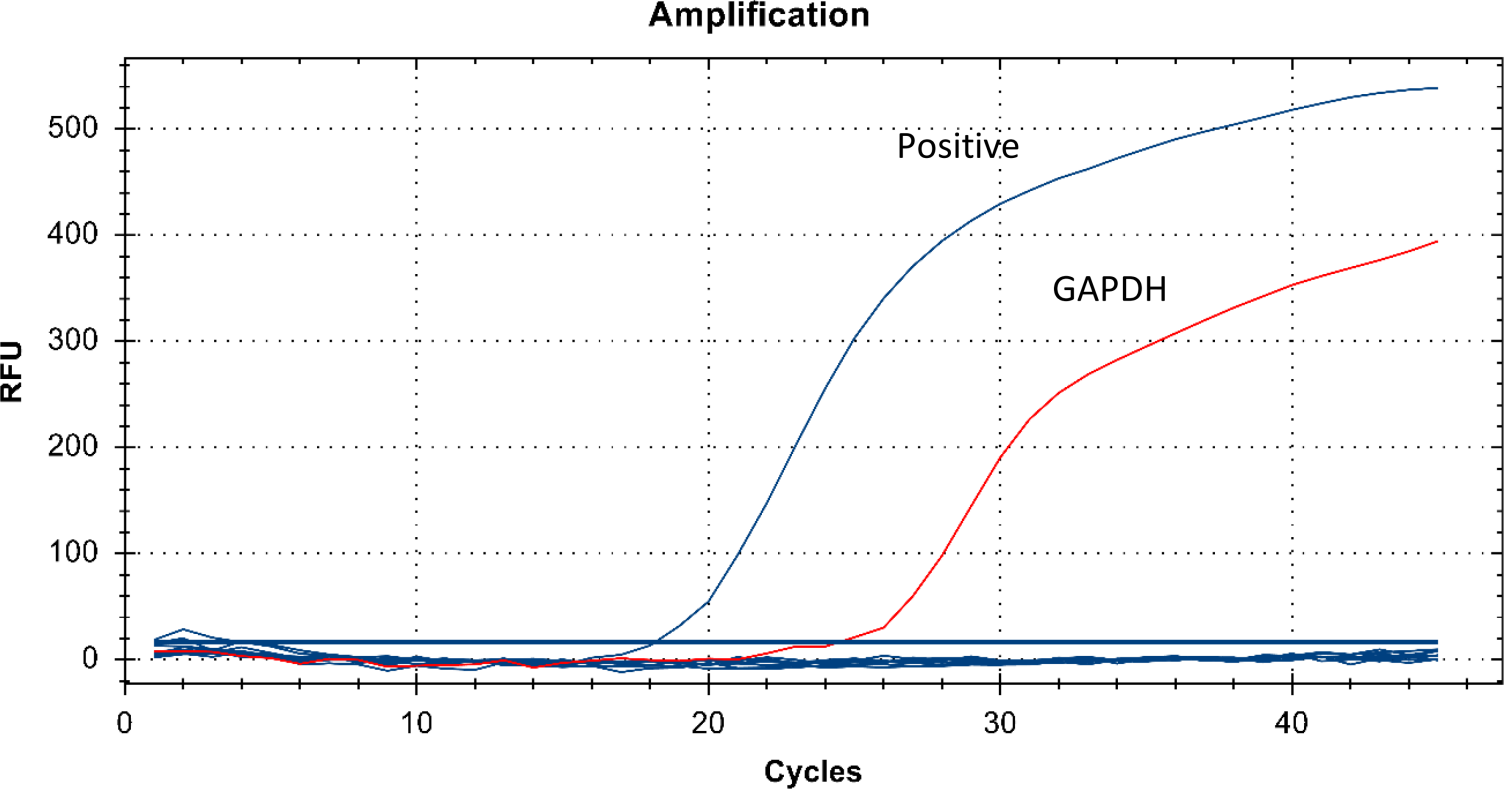
One-tube nested qPCR specificity validation. Amplification curves of positive standards (blue) and the remaining six pathogens including *Escherichia coli*, *Bacillus subtilis*, *Pseudomonas aeruginosa*, *Staphylococcus aureus*, *Streptococcus pneumoniae*, and *Haemophilus influenzae* were all negative with no amplification curve. Red amplification curves represent *GAPDH* (internal quality control).

The specificity of the one-tube nested qPCR assay was then validated. Samples positive for *Brucella* infection and six common pathogens, namely *E. coli*, *Bacillus subtilis*, *Pseudomonas aeruginosa*, *Staphylococcus aureus*, *Streptococcus pneumoniae*, and *Haemophilus influenzae* were used for the assay in this study. The results showed good specificity for the one-tube nested qPCR, with only *Brucella* (positive sample) showing an amplification curve (Fig. 2).

**Fig 3:**
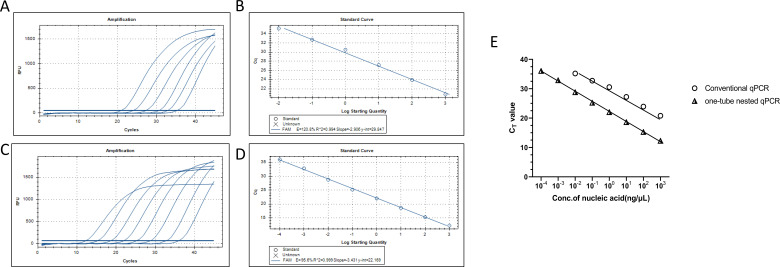
Comparison of analytical sensitivity between one-tube nested qPCR and conventional qPCR analysis. In this study, the *Brucella* genomic DNA was diluted in a 10-fold gradient from 1 × 10^3^ ng/µL to 1 × 10^−4^ ng/µL and subjected to one-tube nested qPCR and conventional qPCR tests. (A, B) Analytical sensitivity and standard curve of conventional qPCR analysis. (C, D) Analytical sensitivity and standard curve of one-tube nested qPCR analysis. (E) Comparison of analytical sensitivity between one-tube nested qPCR and normal qPCR analysis.

In addition, we measured the analytical sensitivity of one-tube nested qPCR by diluting the *Brucella* genomic DNA in a gradient of 1 × 10^3^ ng/µL to 1 × 10^−4^ ng/µL. The results showed that the detection limit of the one-tube nested qPCR was 10^2^ times higher than that of conventional qPCR, and its analytical sensitivity was 100 fg/μL (Fig. 3A and B). The standard curve was used to show average slope values in this assay (−3.431 for one-tube nested qPCR and −2.906 for conventional qPCR). The primer amplification efficiency (AE) of qPCR was 95.6% for the one-tube nested qPCR assay and 120.8% for the conventional qPCR assay, based on the equation AE = (10^−1/slope^ − 1) (Fig. 3C and D). Compared to those with qPCR (CT values from 20.79 to 35.18), the one-tube nested qPCR results produced amplification curves with relatively lower CT values (12.25 to 36.04), indicating that effective amplification in the one-tube nested qPCR assay might increase sensitivity (Fig. 3E).

### Repeatability and reproducibility

To evaluate the reproducibility of the one-tube nested qPCR, *Brucella* genomic DNA of 10^3^ ng/µL, 10^2^ ng/µL, and 10 ng/µL was used in the study for intra-assay and inter-assay studies. The intra-assay and inter-assay coefficients of variation ranged from 0.61% to 1.13% and 0.42% to 3.30%, respectively (Table 2).

**TABLE 2:** Repeatability and reproducibility of one-tube nested qPCR

	Intra-assay		Inter-assay	
*Brucella* genomic DNA (ng/μL)	Mean ± SD^*a*^	CV (%)	Mean ± SD	CV (%)
10^1^	19.00 ± 0.19	0.97	19.58 ± 0.33	1.60
10^2^	15.52 ± 0.10	0.61	15.55 ± 0.07	0.42
10^3^	11.05 ± 0.13	1.13	11.57 ± 0.45	3.30

^*a*^ SD: standard deviation.

### Comparison of clinical performance between the one-tube nested qPCR and conventional qPCR

A total of 250 samples were included in this study: 50 clinical *Brucella* isolates, 145 whole blood samples from patients with brucellosis, and 55 whole blood samples from patients with *E. coli*, *Bacillus subtilis*, *Pseudomonas aeruginosa*, *Staphylococcus aureus*, and *Streptococcus pneumoniae* infections. All samples were tested simultaneously using blood culture, RBPT, one-tube nested qPCR, and conventional qPCR. The results indicate that 100% of the 50 clinical isolates were positively detected using both one-tube nested qPCR and conventional qPCR. In contrast, all 55 whole-blood samples obtained from individuals infected with other pathogens tested negative, ultimately indicating a specificity rate of 100%.

In addition, we used the SPSS 20.0 statistical software to determine kappa values for 200 clinical samples, in order to assess the accuracy of each test method in comparison with clinical diagnostic results. Our results indicate that, when compared to other assays, the one-tube nested qPCR established in this study demonstrated 100% specificity, up to 98.62% sensitivity, and a kappa value of 0.975, representing a high level of consistency with clinical diagnostic results. In contrast, while the RBPT proved to be highly sensitive, it had relatively poor specificity (Table 3). Furthermore, we performed an analysis of the conventional qPCR versus the one-tube nested qPCR using the receiver operating curve, which showed that the one-tube nested qPCR had superior detection performance, with all internal quality controls (GAPDH) effectively amplified (Fig. S3 and Fig.5).

**TABLE 3:** Comparison of the performance of blood cultures, RBPT, conventional qPCR, and one-tube nested qPCR in detecting brucellosis patients (*n* = 145) and patients infected with other pathogens (*n* = 55)

		Brucellosis patients (*n* = 145）	Non-patients (*n* = 55)	Specificity (%)	Sensitivity (%)	Kappa value
Blood culture	Positive	16	0	100.0	11.03	0.064
	Negative	129	55			
RBPT	Positive	137	2	96.36	94.48	0.879
	Negative	8	53			
qPCR	Positive	136	0	100.0	93.79	0.893
	Negative	9	55			
One-tube nested qPCR	Positive	143	0	100.0	98.62	0.975
	Negative	2	55			

**Fig 4:**
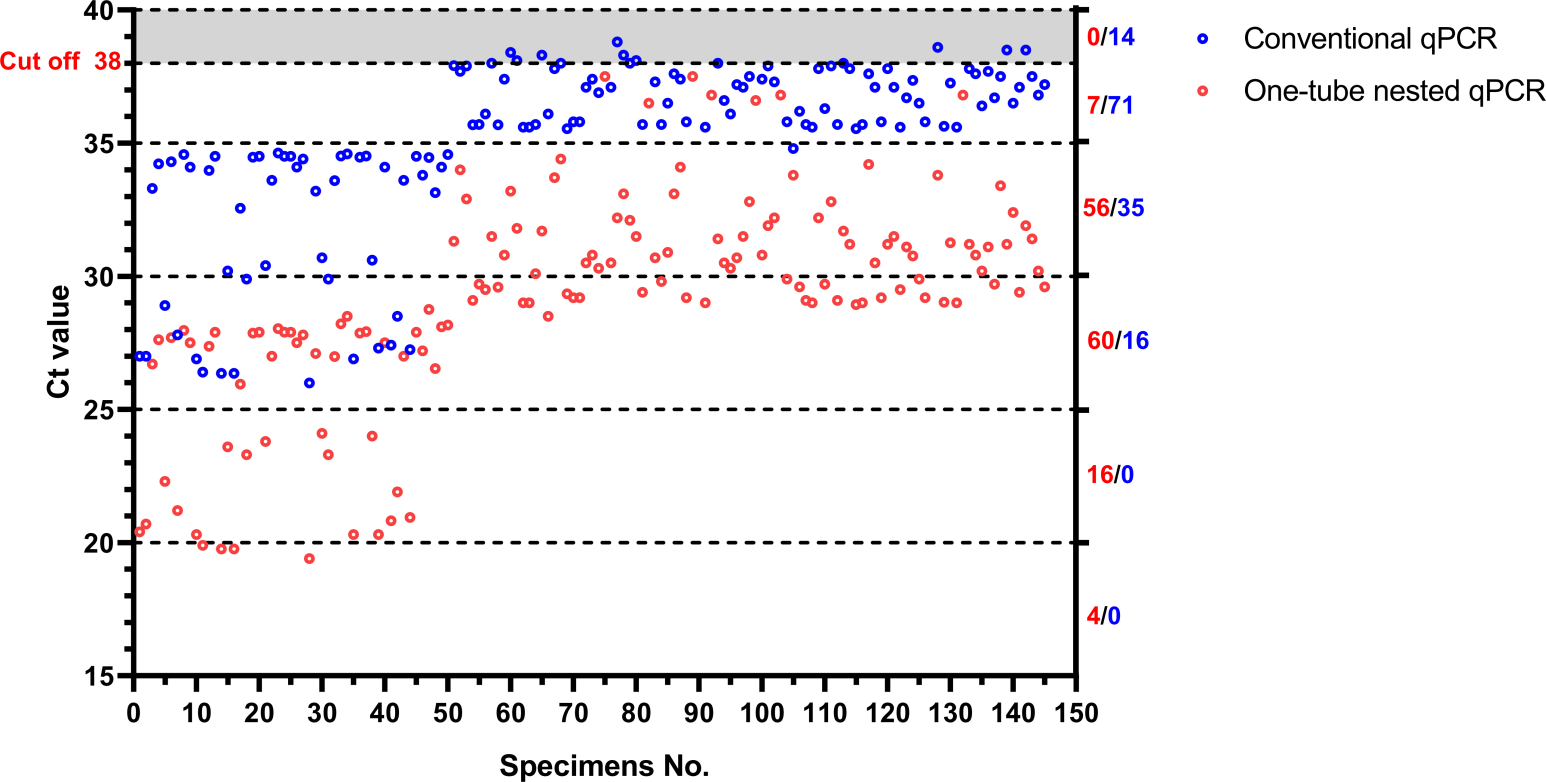
The distribution of number of specimens with different values analyzed by conventional qPCR and one-tube nested qPCR. A total of 145 clinical whole-blood samples, all from patients with brucellosis, were included in the study. Both one-tube nested qPCR assay and conventional qPCR test were performed. The *X*-axis represents the test specimen number, the left *Y*-axis represents the value of the test specimen, and the right *Y*-axis represents the number of one-tube nested qPCR to conventional qPCR positive samples. Results of the one-tube nested qPCR and conventional qPCR assays are indicated by red circles/red font and blue circles/blue font, respectively, in the scatter plot.

After conducting an in-depth analysis of 145 clinical samples, Fig. 4 illustrates the distribution of sample sizes using conventional qPCR and one-tube nested qPCR with varying CT values. Out of the total samples analyzed, 58.6% with a CT > 35 were detected through conventional qPCR. However, the accuracy of detection through qPCR remarkably decreased at a CT > 38, which is typically considered as the cut-off value for qPCR assays. Therefore, using a CT = 38 as the cut-off value, the detection rate of conventional qPCR was 84.1%, while the detection rate of one-tube nested qPCR was 98.6%. This result indicates the advantage of one-tube nested qPCR assays in detecting samples with a low *Brucella* DNA load, as it can decrease the average CT value by 6.4.

## DISCUSSION

Although the gold standard to diagnose brucellosis is the isolation of *Brucella* by culturing samples from blood, bone marrow, or other tissues, the clinical isolation rate from human samples is often relatively low (18, 27). PCR technology, such as nested PCR, quantitative real-time PCR, and a number of other methods, has become the mainstream method for the early and rapid screening of *Brucella*. Several studies have confirmed that the sensitivity of qPCR for the testing of brucellosis is 91.9%, which is a significant improvement over the sensitivity of the culture methods (12). However, the traditional qPCR technique has many drawbacks, particularly the low sensitivity to low loads and the inability to meet the needs of current clinical testing. In recent years, one-tube nested qPCR has been used for the detection of enterovirus, *Mycobacterium tuberculosis*, syncytial virus, and human metapneumovirus owing to its unique advantages (28 – 30). However, no studies have reported on its application for *Brucella* detection and only one literature has reported a semi-nested polymerase chain reaction method for the detection of *Brucella* in soft cheese. Therefore, this study aimed to establish a rapid and highly sensitive one-tube nested qPCR assay that is expected to improve the detection rate of *Brucella* DNA low-load samples and provide a new solution for the clinical diagnosis of brucellosis. We established a rapid and highly sensitive one-tube nested qPCR method for *Brucella* detection using *bcsp31* as the target gene, combining the advantages of general fluorescence qPCR and nested PCR.

**Fig 5:**
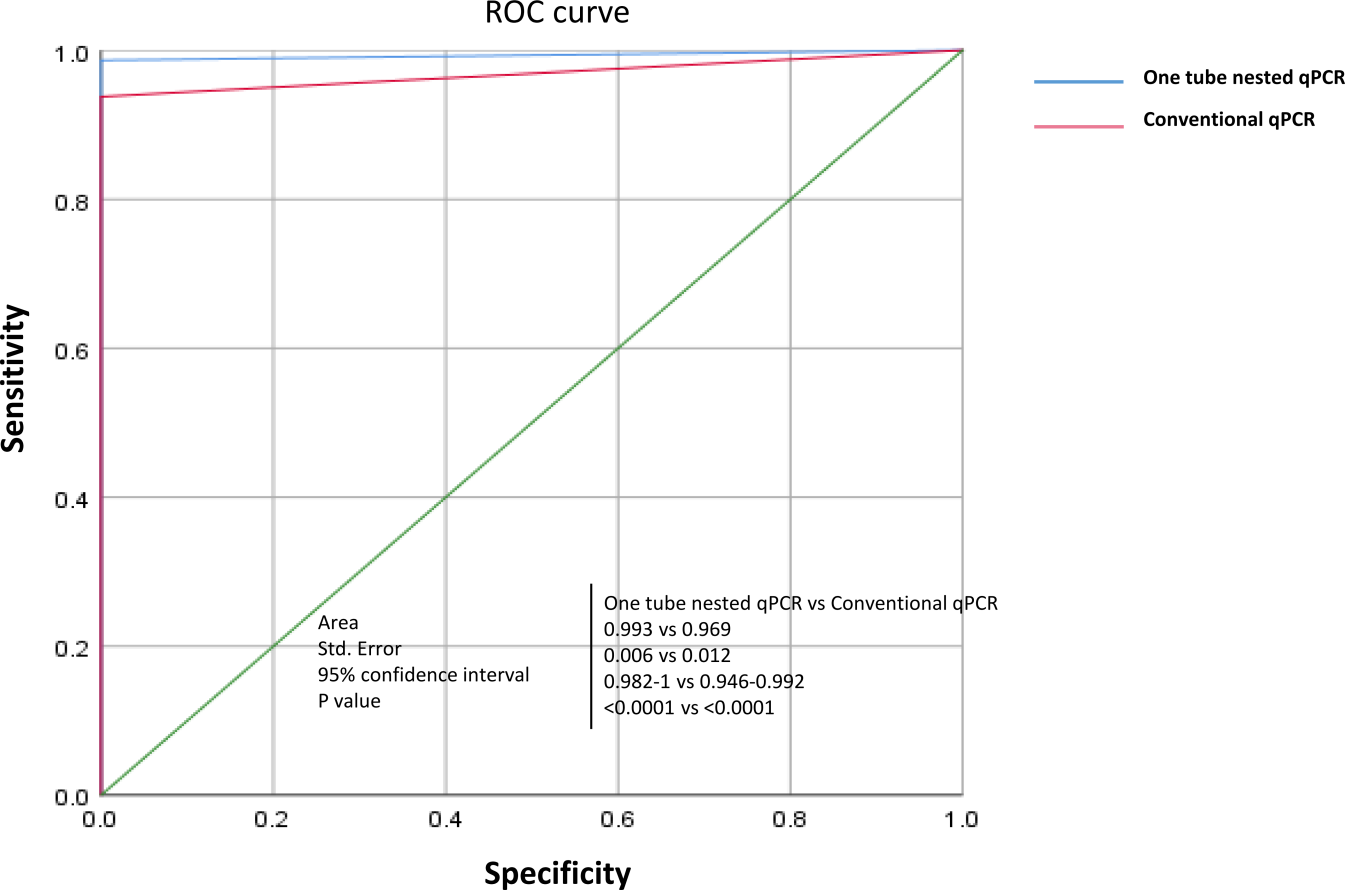
The receiver operating characteristic curve. When the specificity was 100%, the maximum sensitivity of one-tube nested qPCR was 98.6% and the maximum sensitivity of the conventional qPCR was 93.8%, against which the cutoff values were 38.75 and 39.4, respectively.

Two-step nested qPCR has been reported to be more sensitive than qRT-PCR assays (31, 32). However, it has the natural disadvantage of increasing the risk of the cross-contamination of test samples with amplicons from positive samples at the end of the first round of amplification and the transfer of amplicons for the second round of amplification. In the present study, primers and probes were carefully designed to maximize the annealing temperature difference between the outer and inner primer sets, which allowed for successful one-step nested amplification using temperature-switched PCR (TSP). In the in-house assays, the annealing temperatures of the outer and inner primers were optimized, and the highest amplification efficiency was achieved when there was a 10°C difference (68°C versus 58°C), and this TSP strategy was similar to that reported in previous studies (33). In addition, we have performed a comparative study of the analytical sensitivity of one-tube nested qPCR and conventional qPCR and showed that one-tube nested qPCR is approximately 100-fold more sensitive than conventional qPCR. Furthermore, the performance in clinical sample testing confirmed the high sensitivity of one-tube nested qPCR (98.6%) compared with that of conventional qPCR (93.8%) (Fig. 5). The results of the conventional qPCR assay are thus consistent with those of previous studies (26, 34).

To fully evaluate the value of the one-tube nested qPCR for detection of brucellosis, we performed a simple retrospective analysis of previous studies (Table 4). Although SYBR Green-based PCR protocols have demonstrated high sensitivity, relatively low cost, and ease of use for *Brucella* testing, their low specificity due to nonspecific binding of SYBR Green to DNA cannot be ignored. Therefore, TaqMan probe-based protocols have gained increasing acceptance to improve the sensitivity, specificity, and reproducibility of *Brucella* detection. Recently, several TaqMan probe-based *Brucella* detection studies have achieved dramatic improvements in terms of assay, with lower limits of detection as low as to 1–2 genomic copies (Table 4) (35, 36). Whereas previous studies have shown the good performance and analytical sensitivity of in-house assays, the specificity and sensitivity in subsequent assays using clinical specimens have been less than satisfactory; and in some studies, only a small number of clinical specimens or no clinical studies were included (Table 4). The reasons for the differences in the performance of these methods in detecting clinical specimens are multiple, including the acquisition of high-quality DNA templates and differences in the detection methods themselves, such as the primer amplification efficiency and type of probe modification, etc. Similarly, the low sensitivity of all assays for detecting clinical samples with low *Brucella* DNA load leads to clinical misdiagnoses, which endangers the life and health of patients.

**TABLE 4:** Comparison of qPCR protocols used in the current and previous studies

Methods	Target	Sample type	Number of clinical samples	Lowest limit of detection	Sensitivity	Specificity	Species	Reference
SYBR Green-based qPCR	*bcsp31*	Serum	188	10 genome copies	n.a.^*a*^	100	*Brucella*	(18)
	*bcsp31*	Serum	97	n.a.	93.3	91.9	*Brucella*	(12)
	*bcsp31*	Serum	127	5 fg *Brucella* DNA	91.9	95.4	*Brucella*	(37)
	*BMEII0466*	Spondylitis	31	n.a.	93.5	100	*B. melitensis*	(19)
	*IS711*	Aborted fetal stomach	56	11.8 fg *Brucella* DNA	44.6	100	*Brucella*	(20)
	*alkB-IS711*	*B. abortus*	0	2 genome copies	100	83	*B. abortus*	(38)
	*IS711-BMEI1162*	*B. melitensis*	0	112 genome copies	100	100	*B. melitensis*	(39)
	*bcsp31*	Serum	188	10 genome copies	n.a.	100	*Brucella*	(18)
	*IS711*	Whole-blood	530	1E + 01 cfu/mL to 1E + 08 cfu/mL	93.14	100	*B. melitensis*, *B. abortus*, *B. suis*	(34)
TaqMan-based qPCR	*bcsp31*	Serum	110	10 fg genomic DNA	93.5	98.4	*Brucella*	(21)
	*bcsp31*	Serum	62	2 genome copies	72	100	*Brucella*	(35)
	*Acetyl-CoA C-acetyltransferase*	*B. melitensis* human clinical isolates	120	6.25 genome copies	80	100	*B. melitensis*	(40)
	*bcsp31*	Blood, milk, nasal, vaginal swabs	867	1 genome copy	77.8	100	*Brucella abortus*	(36)
	*alkB-IS711*	*B. abortus*	0	2 genome copies	100	83	*B. abortus*	(38)
	*bcsp31*	Whole-blood	250	10 ng plasmid DNA	93.8	100	*Brucella*	This study
	*IS711*	*B. abortus*	0	10 copies/reaction	n.a.	100	*B. abortus*	(41)
	*bcsp31*			20 copies/reaction				
	*eryC*	Sheep raw milk and pig blood	n.a.	1 genome/reaction	100	n.a.	*Brucella*	(23)
Conventional PCR	*IS711*	Whole-blood	267	n.a.	46.4	100	*B. melitensis*, *B. abortus*, *B. suis*	(42)
	*bcsp31*	Serum	180	n.a.	96	80.7	*Brucella*	(25)
	*IS711*	Serum	180	n.a.	82	80.7	*B. melitensis*, *B. abortus*	
	*IS711*	Aborted fetal stomach	56	11.8 fg genomic DNA	44.6	100	*Brucella*	(20)
	*bcsp31*	Whole-blood, serum	120	n.a.	68.18/56.06	100	*Brucella*	(27)
One-tube nested qPCR	*bcsp31*	Whole-blood	250	100 fg plasmid DNA	98.6	100	*Brucella*	This study

^*a*^ n.a. not applicable.

In this study, we applied the TSP strategy to detect *Brucella* for the first time. The working concentration of each primer, the reaction conditions, and the running conditions of a single-tube nested qPCR were optimized. This allowed for the detection of *Brucella* with high sensitivity and good specificity. It is noteworthy that the one-tube nested qPCR assay can significantly improve the CT value of the assay in our study, which means that those samples with low DNA load that cannot be detected by conventional qPCR could be detected by one-tube nested qPCR. In addition, a comparative study with previous studies showed that our established one-tube nested qPCR performed optimally when using clinical samples with a sensitivity of 98.6% (Table 4). This greatly improves the detection rate and accuracy with clinical samples. Combining the results of previous studies with our data, we recommend using this sensitive one-tube nested qPCR in this study for the testing of *Brucella*.

Our results have demonstrated the superiority of one-tube nested qPCR in aiding the diagnosis of brucellosis. However, our study has some shortcomings. *Brucella* can infect bones and joints, the central nervous system, and the respiratory system, causing a variety of complications. Clinical testing samples are diverse in nature and in this study, we only tested blood samples. Other tissue fluids such as joint exudate, cerebrospinal fluid, and alveolar lavage fluid were not systematically evaluated. Therefore, the sensitivity and specificity of the one-tube nested qPCR when using different samples need to be further investigated; this is an area of ongoing research to better support the early diagnosis of brucellosis and clinical use of testing and to develop optimal diagnostic and therapeutic protocols to improve human life and health.

### Conclusion

In summary, we developed a new, efficient, and highly sensitive qPCR assay, a one-tube nested qPCR for *bcsp31* sequence. This exploits the advantages of both nested PCR and real-time PCR and achieves a qualitative increase in the sensitivity and accuracy of the assay for detecting *Brucella* samples with low DNA loads. This one-tube nested real-time PCR showed superior sensitivity to conventional real-time PCR and has the potential to be widely used as a rapid test for *Brucella* in provincial and municipal areas of China.

## SUPPLEMENTAL MATERIAL

Fig. S1 spectrum.00582-23-s0001.pdf Brucellosis identification.

Fig. S2 spectrum.00582-23-s0002.pdf Optimization of optimal annealing temperature and primer concentration for single tube nested qPCR.

Fig. S3 spectrum.00582-23-s0003.pdf One-tube nested qPCR amplification curve for detection of clinical samples.

Supplemental legends spectrum.00582-23-s0004.docx Legends for Figures S1 to S3.

ASM does not own the copyrights to Supplemental Material that may be linked to, or accessed through, an article. The authors have granted ASM a non-exclusive, world-wide license to publish the Supplemental Material files. Please contact the corresponding author directly for reuse.
