# Supplementary material for: A novel, highly sensitive, one-tube nested quantitative real-time PCR for Brucella in human blood samples
Source: Microbiol Spectr. 2023 Oct 4;11(6):e00582-23. doi: 10.1128/spectrum.00582-23 (PMC10714840; doi:10.1128/spectrum.00582-23)
Supplement: Fig. S1 — Brucellosis identification. [file spectrum.00582-23-s0001.pdf]

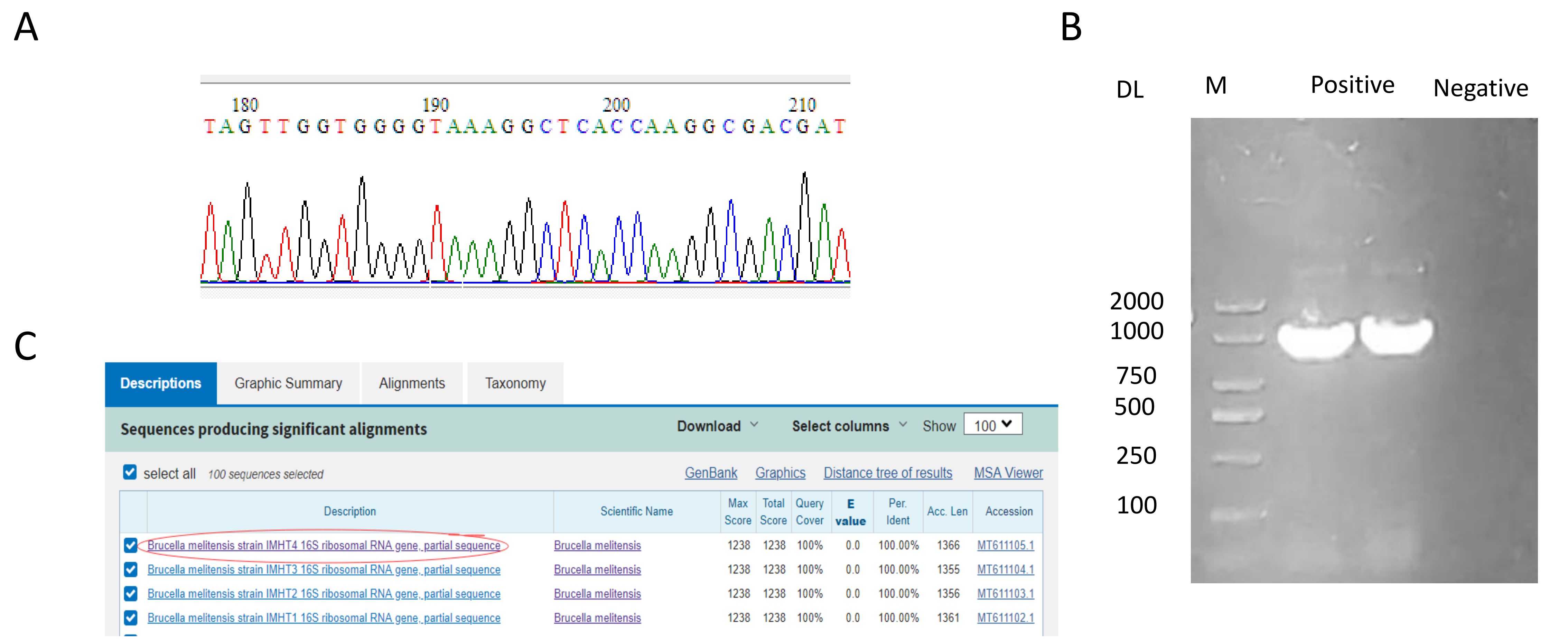
A
180
190
200
210
TAGTTGGTGGGGTAAAGGCTCACCAAGGCGACGAT
B
DL
M
Positive
Negative
2000
1000
750
500
250
100
C
Descriptions
Graphic Summary
Alignments
Taxonomy
Sequences producing significant alignments
Download
Select columns
Show 100
select all 100 sequences selected
GenBank
Graphics
Distance tree of results
MSA Viewer
Description | Scientific Name | Max Score | Total Score | Query Cover | E value | Per. Ident | Acc. Len | Accession
Brucella melitensis strain IMHT4 16S ribosomal RNA gene, partial sequence | Brucella melitensis | 1238 | 1238 | 100% | 0.0 | 100.00% | 1366 | MT611105.1
Brucella melitensis strain IMHT3 16S ribosomal RNA gene, partial sequence | Brucella melitensis | 1238 | 1238 | 100% | 0.0 | 100.00% | 1355 | MT611104.1
Brucella melitensis strain IMHT2 16S ribosomal RNA gene, partial sequence | Brucella melitensis | 1238 | 1238 | 100% | 0.0 | 100.00% | 1356 | MT611103.1
Brucella melitensis strain IMHT1 16S ribosomal RNA gene, partial sequence | Brucella melitensis | 1238 | 1238 | 100% | 0.0 | 100.00% | 1361 | MT611102.1
