# Supplementary figures and images for: A novel, highly sensitive, one-tube nested quantitative real-time PCR for Brucella in human blood samples
Source: Microbiol Spectr. 2023 Oct 4;11(6):e00582-23. doi: 10.1128/spectrum.00582-23 (PMC10714840; doi:10.1128/spectrum.00582-23)

A

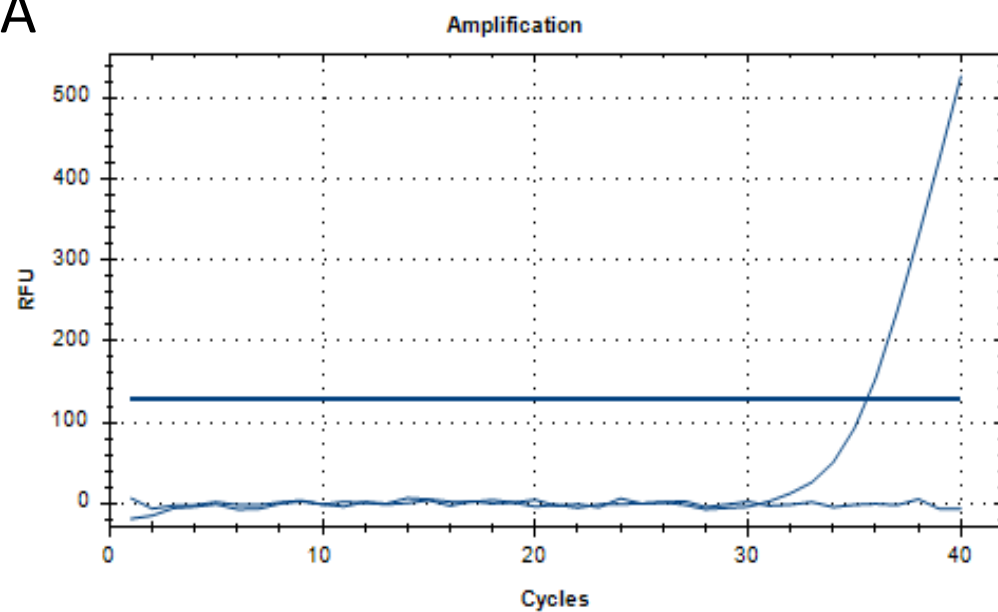

B

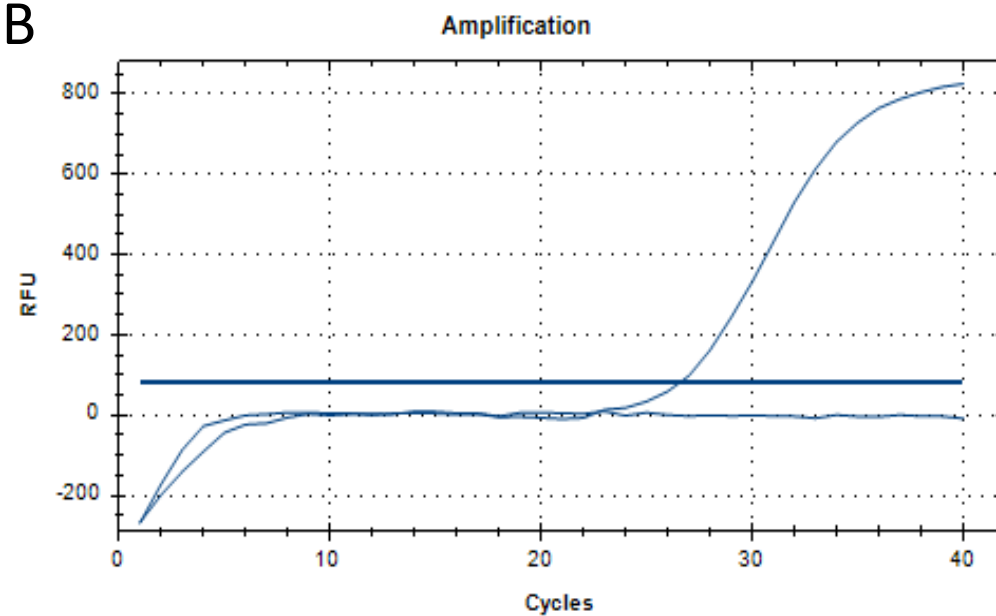

C

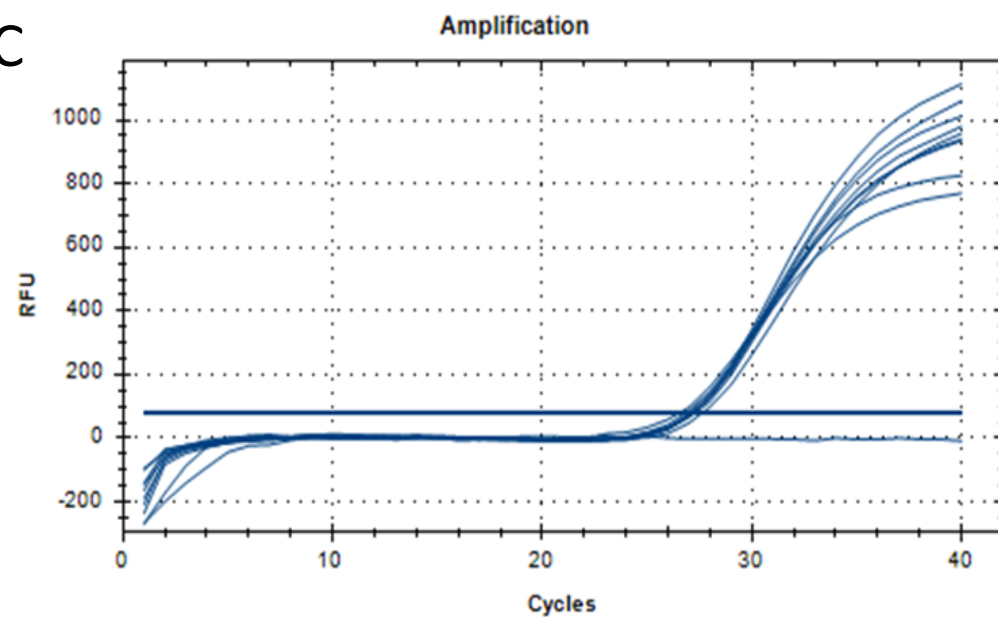

Supplement: Fig. S2 — Optimization of optimal annealing temperature and primer concentration for single tube nested qPCR. [file spectrum.00582-23-s0002.pdf]

# Amplification

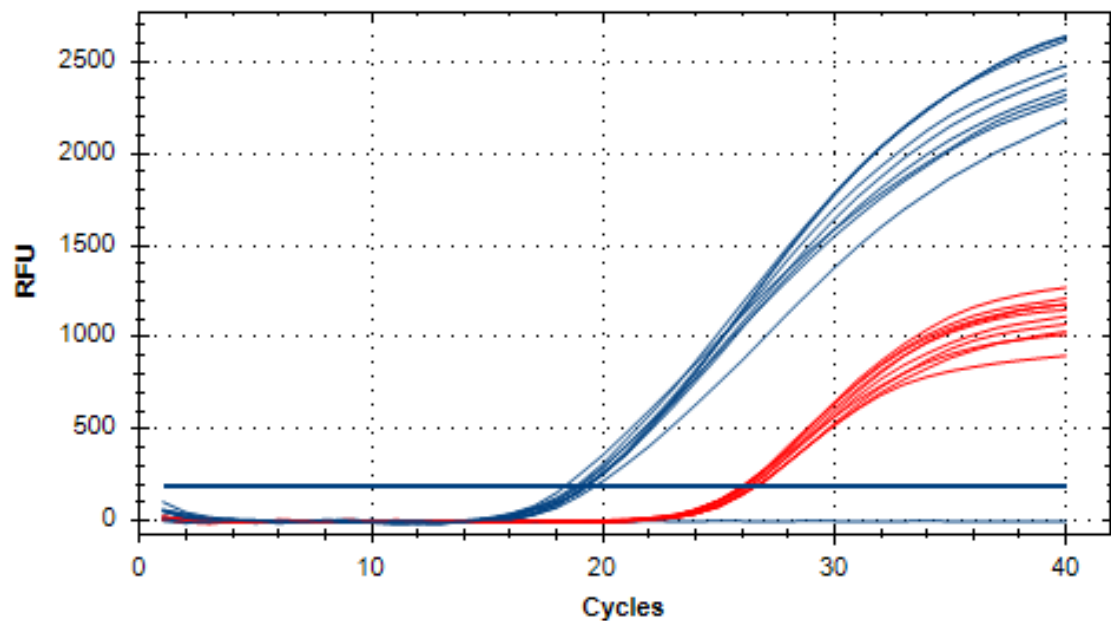

Supplement: Fig. S3 — One-tube nested qPCR amplification curve for detection of clinical samples. [file spectrum.00582-23-s0003.pdf]
