# Supplementary material for: A novel, highly sensitive, one-tube nested quantitative real-time PCR for Brucella in human blood samples
Source: Microbiol Spectr. 2023 Oct 4;11(6):e00582-23. doi: 10.1128/spectrum.00582-23 (PMC10714840; doi:10.1128/spectrum.00582-23)
Supplement: Supplemental legends — Legends for Figures S1 to S3. [file spectrum.00582-23-s0004.docx]

**Figure S1. Brucellosis Identification. Amplification and identification of Brucella isolates using 16S rRNA gene-specific primers.** (A) 2% agarose gel electrophoresis, target gene amplification fragment size of about 900 bp. (B) Singer sequencing of samples was performed using 27F/1492R primer pair to obtain 16S rRNA sequences. (C) NCBI Blast revealed that the sample was Brucella, and the similarity was 100% to Brucella melitensis strain IMHT4.

**Figure S2. Optimization of optimal annealing temperature and primer concentration for one-tube nested qPCR.** (A) Plot with annealing temperature of 66°C and value of 35.6. (B) Plot with annealing temperature of 68°C and value of 26.2. (C) Optimization of primer concentration for qPCR. The final primer concentrations of 240 nmol/L, 280 nmol/L, 320 nmol/L, 360 nmol/L, 400 nmol/L, 440 nmol/L, 480 nmol/L, 520 nmol/L and 560 nmol/L were set for detection. Their s ranged from 26.01 to 27.68 with no significant difference. To save the cost of the assay, 400 nmol/L was used as the optimal primer concentration.

**Figure S3. One-tube nested qPCR amplification curve for clinical sample.** One-tube nested qPCR amplification curves obtained for 10 cases selected from 145 clinical samples. Blue amplification curves represent Brucella and red amplification curves represent GAPDH (internal quality control). RFU relative fluorescence units.
